# The platelet-to-high-density-lipoprotein-cholesterol ratio: a promising biomarker for predicting major adverse cardiovascular events in the Chinese community-based population

**DOI:** 10.3389/fcvm.2026.1868836

**Published:** 2026-10-14

**Authors:** Lei Dong, Long Zhang, Fangfang Fan, Yan Zhang, Jianping Li

**Affiliations:** 1 Department of Cardiology, Peking University First Hospital, Beijing, China; 2 Institute of Cardiovascular Disease, Peking University First hospital, Beijing, China

**Keywords:** acute myocardial infarction, biomarkers, Chinese community-based population, major adverse cardiovascular events, platelet to high density lipoprotein-cholesterol ratio

## Abstract

**Background:**

The platelet-to-high-density-lipoprotein-cholesterol ratio (PHR), a composite index integrating platelet count and HDL-C concentration, has recently attracted attention as a potential indicator of inflammatory and metabolic status. However, evidence regarding its relationship with major adverse cardiovascular events (MACE) is still limited. Therefore, using data from a prospective community-based cohort, we evaluated whether baseline PHR was associated with the occurrence of MACE during 10 years of follow-up.

**Methods:**

A total of 8,184 eligible subjects were included in our analysis. The primary outcome was MACE, and the secondary outcome was acute myocardial infarction (AMI), stroke, and CV mortality over 10 years. The PHR was calculated as the ratio of platelets to high-density-lipoprotein-cholesterol (HDL-C). Associations between baseline PHR and subsequent MACE were examined using multivariable Cox proportional hazards models, with effect estimates reported as hazard ratios (HRs) and corresponding 95% confidence intervals (CIs).

**Results:**

Of all participants, the mean age was 56.4 ± 8.9 years, and 2,989 (36.5%) were men. During a mean 10-year follow-up, a total of 853 (10.4%) cases of MACE, 165 (2.0%) cases of AMI, 673 (8.2%) cases of stroke, and 143 (1.7%) cases of CV mortality were recorded. After multivariable adjustment, individuals in the highest PHR quartile showed a greater risk of MACE than those in the lowest quartile (HR: 1.37; 95% CI: 1.12–1.69). A similar association was observed for AMI, with the highest quartile having more than twice the risk of the lowest quartile (HR: 2.44; 95% CI: 1.43–4.18). In contrast, the corresponding associations with stroke (HR: 1.27; 95% CI: 1.00–1.60) and CV mortality (HR: 1.07; 95% CI: 0.65–1.77) were not statistically significant.

**Conclusions:**

These findings indicate that higher PHR is associated with an increased long-term risk of MACE and AMI among Chinese community-dwelling adults, suggesting that PHR may serve as a potential biomarker associated with cardiovascular risk.

## Introduction

The platelet-to-high-density-lipoprotein-cholesterol ratio (PHR), calculated from platelet count and HDL-C concentration, has been proposed as a novel composite indicator reflecting inflammatory and metabolic alterations (1). Lu et al., using data from the 2017–2020 National Health and Nutrition Examination Survey (NHANES), reported that PHR was associated with non-alcoholic fatty liver disease (NAFLD) and hepatic fibrosis, suggesting its potential value as a biomarker for these conditions (2). In that study, the relationship between PHR and NAFLD appeared to vary across different PHR ranges: when PHR was below 181, lower values were associated with a decreased likelihood of NAFLD. For hepatic fibrosis, an inverse association was observed when PHR was above 162, with lower PHR values corresponding to a higher risk. These findings indicate that PHR may reflect both metabolic abnormalities and inflammatory status (3).

Metabolic syndrome and inflammation are identified risk factors for cardiovascular diseases (4, 5). Taken together, these observations raise the possibility that PHR could help recognize individuals who are more susceptible to major adverse cardiovascular events (MACE) before clinical events occur, thereby allowing earlier preventive strategies. Nevertheless, the relationship between PHR and future MACE has not been sufficiently investigated. To address these research gaps, we performed this longitudinal cohort study to investigate whether PHR levels were associated with the risk of MACE over a 10-year follow-up in a Chinese community-based population.

## Methods

### Ethics

This study was approved by the ethics committee of Peking University First Hospital, and each participant provided written informed consent.

### Study population

The study population was derived from a cohort survey focused on atherosclerosis, which included 9,540 community-dwelling residents aged 40 years or older. The survey was conducted in Beijing, China, from December 2011 to April 2012. After excluding participants who missed data (*n* = 408) and had previously been prescribed lipid-lowering medication (*n* = 948), 8,184 participants were included (Figure 1).

**Figure 1:**
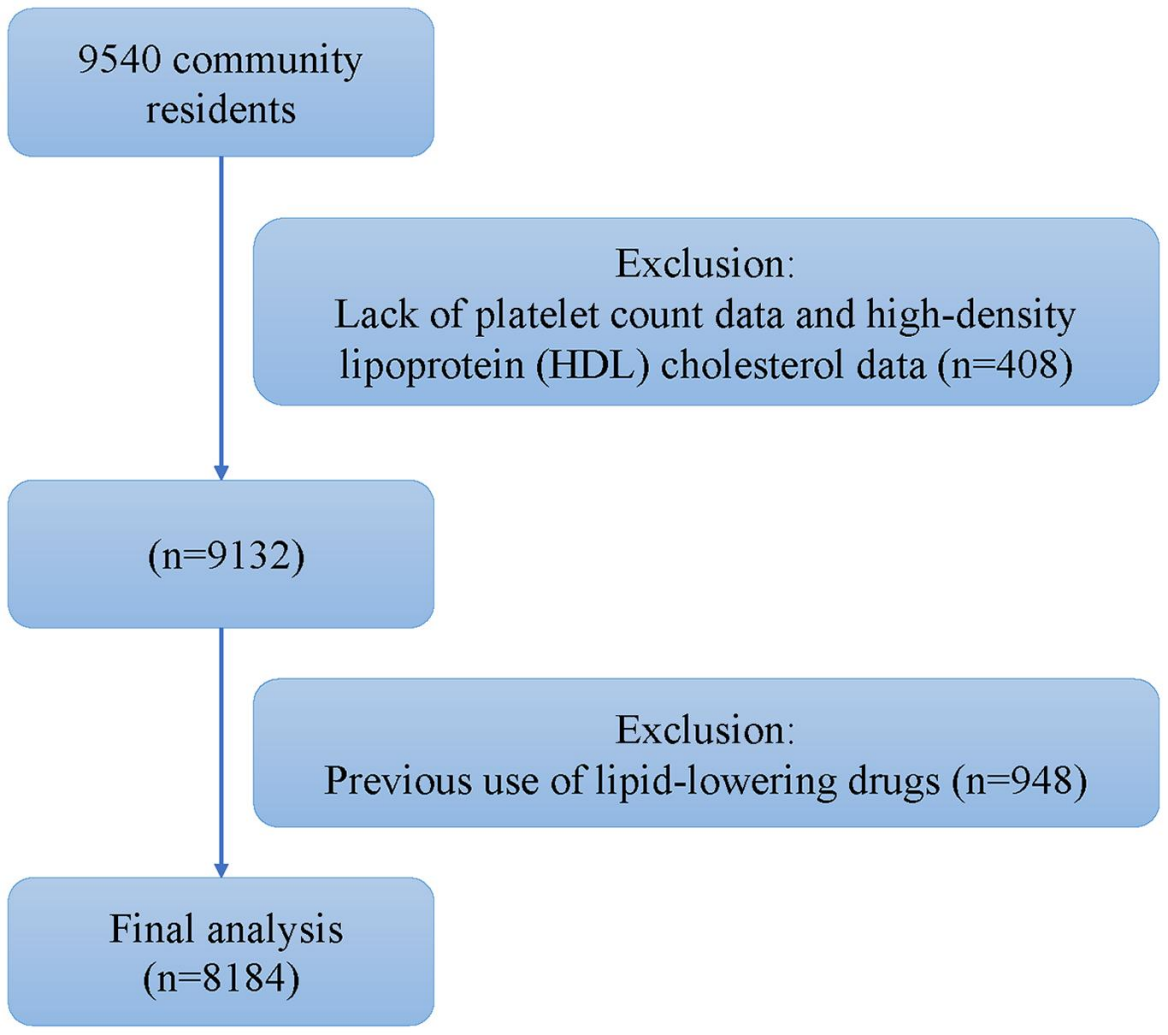
Study participant enrollment process.

### Data collection

At baseline, trained research staff collected participant information following predefined standard procedures. A structured and previously validated questionnaire was used to obtain data on demographic and socioeconomic characteristics, education, occupation, dietary patterns, lifestyle and health behaviors, and medical history (6). Body mass index (BMI) was determined by dividing body weight in kilograms by the square of height in meters. Baseline serum triglycerides (TG), high-density lipoprotein cholesterol (HDL-C), and low-density lipoprotein cholesterol (LDL-C) were measured from collected blood samples. Laboratory testing at baseline was performed with a Roche C8000 automatic analyzer (Basel, Switzerland). We apply the Chronic Kidney Disease Epidemiology Collaboration (7) to calculate estimated glomerular filtration rate (eGFR). Participants were classified as current smokers if they had smoked at least one cigarette per day for 6 months or longer. Current drinking was defined as alcohol consumption at least once weekly for a minimum of 6 months. Platelet counts at baseline were determined in the clinical laboratory of Peking University Shougang Hospital using a BC-3000 automated hematology analyzer (Mindray Medical International, Shenzhen, China). PHR was derived from the same blood sample by dividing the platelet count by the HDL-C concentration, expressed as platelet count (10⁹/L)/HDL-C (mmol/L) (8).

### Outcome measure

The outcomes were classified according to the 10th Revision of the International Classification of Diseases (ICD-10). The primary outcome of the study was major adverse cardiovascular events (MACE), defined as a composite of cardiovascular (CV) mortality (ICD-10 codes: I00–I99), stroke (including transient ischemic attack) (I60, I61, I63, I64), and acute myocardial infarction (AMI) (I21.0, I21.1, I21.2, I21.3, I21.4, I21.9, I22.0, I22.1, I22.8, I22.9). The secondary outcomes included AMI, stroke, and CV mortality. The outcome data for this study were ascertained through linkage of participants' identification information with an external database. Information regarding non-fatal myocardial infarction and stroke events was obtained from the Beijing Municipal Health Commission's Inpatient Medical Records Homepage System. The information of death was obtained from the Chinese Center for Disease Control and Prevention-National Mortality Surveillance System (9), with a deadline of December 31, 2021.

### Statistical analysis

Continuous variables are summarized as mean ± standard deviation (SD) when normally distributed and as median with interquartile range (IQR) when skewed. The normality of continuous variables was assessed using the Shapiro–Wilk test. Categorical variables are expressed as numbers and percentages. For comparisons across PHR groups, normally distributed continuous variables were evaluated by one-way analysis of variance (ANOVA), whereas skewed variables were compared using the Kruskal–Wallis test. Differences in categorical variables were tested with the chi-square (*χ*²) test. Participants were categorized into quartiles according to the 25th, 50th, and 75th percentiles of the baseline PHR distribution. Kaplan–Meier survival curves were constructed according to PHR quartiles, and differences between groups were compared using the log-rank test.

The relationships of PHR with MACE and each secondary endpoint were evaluated using multivariable Cox proportional hazards models. Results from these models are presented as hazard ratios (HRs) with 95% confidence intervals (CIs).

We developed three Cox regression models with different levels of covariate adjustment. Model 1 included PHR only and was used as the unadjusted model. Model 2 included additional adjustment for age and sex. Model 3 was further adjusted for current smoking, current drinking, diabetes history, hypertension, use of hypoglycemic medication, use of antihypertensive medication, BMI, eGFR, LDL-C, and TG. Restricted cubic spline models were fitted to explore the potential linear and nonlinear dose–response associations of PHR with MACE and AMI.

Additional stratified analyses were conducted according to sex, age (<65 or ≥65 years), smoking status, drinking status, diabetes, hypertension, and BMI (<28 or ≥28 kg/m²). Potential effect modification was assessed by testing interaction terms between PHR and each stratification variable.

All statistical analyses were conducted using R software, version 4.3.2. A two-sided *P* value <0.05 was considered statistically significant.

## Results

### Baseline characteristics of study participants

Of all participants, the mean age was 56.4 ± 8.9 years, and 2,989 (36.5%) were men. During a mean 10-year follow-up, a total of 853 (10.4%) cases of MACE, 165 (2.0%) cases of AMI, 673 (8.2%) cases of stroke, and 143 (1.7%) cases of CV mortality were recorded. Baseline sociodemographic characteristics and clinical covariates stratified by PHR quartiles are summarized in Table 1. The PHR cut-off values for Q1 to Q4 were <116.4, 116.4–147.5, 147.5–184.1, and ≥184.1, respectively. Most baseline variables differed significantly across PHR quartiles, whereas current drinking and hypoglycemic medication use showed no significant differences. Compared with participants in the lower PHR quartiles, those in Q4 were more frequently male and current smokers, and had a higher prevalence of diabetes and hypertension, greater use of antihypertensive medication, and higher levels of TG, BMI, and eGFR (all *P* < 0.05).

**Table 1:** Baseline characteristics of study participants.

Characteristics	Total (*N* = 8,184)	Q1 (*N* = 2,048) (<116.4)	Q2 (*N* = 2,044) (116.4–147.5)	Q3 (*N* = 2,046) (147.5–184.1)	Q4 (*N* = 2,046) (≥184.1)	*P*-value
Sex, *N* (%)						<.001
Male	2,989 (36.5%)	619 (30.2%)	727 (35.6%)	770 (37.6%)	873 (42.7%)	
Female	5,195 (63.5%)	1,429 (69.8%)	1,317 (64.4%)	1,276 (62.4%)	1,173 (57.3%)	
Age, median (IQR)	55.0 (50.0–61.0)	56.0 (51.0–63.0)	56.0 (51.0–62.0)	55.0 (50.0–61.0)	54.0 (49.0–59.0)	<0.001
Current smoking, *N* (%)	1,600 (19.5%)	277 (13.5%)	338 (16.5%)	427 (20.9%)	558 (27.3%)	<0.001
Current drinking, *N* (%)	1,968 (24.0%)	475 (23.2%)	496 (24.3%)	500 (24.4%)	497 (24.3%)	0.775
Hypertension, *N* (%)	3,789 (46.3%)	844 (41.2%)	893 (43.7%)	997 (48.7%)	1,055 (51.6%)	<0.001
Diabetes, *N* (%)	1,864 (22.8%)	405 (19.8%)	433 (21.2%)	494 (24.1%)	532 (26%)	<0.001
Antihypertensive drug, *N* (%)	2,240 (27.4%)	482 (23.5%)	510 (25%)	588 (28.7%)	660 (32.3%)	<0.001
Hypoglycemic drug, *N* (%)	728 (8.9%)	171 (8.3%)	165 (8.1%)	195 (9.5%)	197 (9.6%)	0.184
LDL-C, median (IQR)	3.2 (2.7–3.8)	3.1 (2.6–3.6)	3.2 (2.8–3.8)	3.3 (2.8–3.8)	3.3 (2.8–3.8)	<0.001
TG, median (IQR)	1.3 (0.9–1.8)	1.0 (0.7–1.3)	1.2 (0.9–1.6)	1.4 (1.0–1.9)	1.8 (1.3–2.5)	<0.001
BMI, median (IQR)	25.7 (23.6–28.0)	24.4 (22.2–26.6)	25.4 (23.4–27.6)	26.1 (24.1–28.4)	26.9 (24.9–29.1)	<0.001
eGFR, median (IQR)	97.5 (88.4–103.8)	96.8 (87.9–103.2)	97.1 (87.8–103.6)	97.7 (88.7–104.3)	98.4 (89.2–104.8)	<0.001

LDL-C, low-density lipoprotein cholesterol; TG, triglyceride; BMI, body mass index; eGFR, estimated glomerular filtration rate; PHR, platelet to high density lipoprotein-cholesterol ratio;.

### Association of PHR with risk of cardiovascular outcomes

To evaluate the independent association between PHR and cardiovascular outcomes, we fitted three Cox regression models with progressively increased covariate adjustment. The corresponding hazard ratios and 95% confidence intervals are presented in Table 2. For these analyses, PHR was categorized into quartiles rather than analyzed solely as a continuous variable. Compared with participants in Q1 of PHR, there was a significantly increased risk of MACE and AMI for participants in Q4 in the fully adjusted model (model 3). The corresponding HR for MACE was 1.37 (95% CI, 1.12–1.69), and the HR for AMI was 2.44 (95% CI, 1.43–4.18). No significant correlations were found between PHR level with the risk of stroke and CV mortality (Table 2). To further characterize the associations of PHR and MACE and AMI, we performed restricted spline analyses using a penalized spline approach. In the fully adjusted model, higher PHR levels were associated with an increased risk of both MACE (Figure 2A) and AMI (Figure 2B).

**Table 2:** Cox regression of the association between PHR with risks of MACE, AMI, stroke and CV mortality in Chinese community-based population.

PHR	No. of Events	Model 1^a^		Model 2^b^		Model 3^c^	
		HR (95%CI)	*P*-value	HR (95%CI)	*P*-value	HR (95%CI)	*P*-value
MACE							
Q1	186	Ref.		Ref.		Ref.	
Q2	191	1.03 (0.84, 1.26)	0.776	1.08 (0.88, 1.33)	0.440	1.03 (0.84, 1.26)	0.797
Q3	220	1.19 (0.98, 1.45)	0.081	1.37 (1.12, 1.66)	0.002	1.18 (0.96, 1.45)	0.125
Q4	256	1.40 (1.16, 1.69)	<0.001	1.71 (1.41, 2.07)	<0.001	1.37 (1.12, 1.69)	0.003
*P* for trend			<0.001		<0.001		<0.001
AMI							
Q1	20	Ref.		Ref.		Ref.	
Q2	40	2.01 (1.17, 3.44)	0.011	2.10 (1.23, 3.60)	0.007	1.87 (1.09, 3.21)	0.024
Q3	46	2.30 (1.36, 3.89)	0.002	2.59 (1.53, 4.39)	<0.001	1.98 (1.16, 3.39)	0.013
Q4	59	2.97 (1.79, 4.93)	<0.001	3.51 (2.11, 5.86)	<0.001	2.44 (1.43, 4.18)	0.001
*P* for trend			<0.001		<0.001		0.022
Stroke							
Q1	153	Ref.		Ref.		Ref.	
Q2	147	0.96 (0.77, 1.20)	0.721	1.01 (0.80, 1.26)	0.952	0.96 (0.76, 1.20)	0.711
Q3	179	1.17 (0.95, 1.46)	0.146	1.34 (1.08, 1.67)	0.007	1.17 (0.93, 1.46)	0.181
Q4	194	1.28 (1.04, 1.58)	0.022	1.56 (1.26, 1.94)	<0.001	1.27 (1.00, 1.60)	0.050
*P* for trend			0.006		<0.001		0.001
CV mortality							
Q1	43	Ref.		Ref.		Ref.	
Q2	38	0.88 (0.57, 1.36)	0.573	1.00 (0.64, 1.54)	0.989	1.00 (0.64, 1.65)	0.999
Q3	30	0.69 (0.43, 1.10)	0.123	0.94 (0.59, 1.51)	0.811	0.88 (0.54, 1.43)	0.599
Q4	32	0.74 (0.46, 1.17)	0.198	1.14 (0.72, 1.81)	0.581	1.07 (0.65, 1.77)	0.783
*P* for trend			0.100		0.686		0.953

MACE, major adverse cardiovascular event; AMI, acute myocardial infarction; CV, cardiovascular; CI, confidence interval; HR, hazard ratio.

a Model 1 was a crude model that only incorporates PHR.

b Model 2 adjusted for age, sex.

c Model 3 adjusted for age, sex, smoke, drink, hypertension, diabetes, antihypertensive drug, hypoglycemic drug, LDL-C, TG, BMI, eGFR.

**Figure 2:**
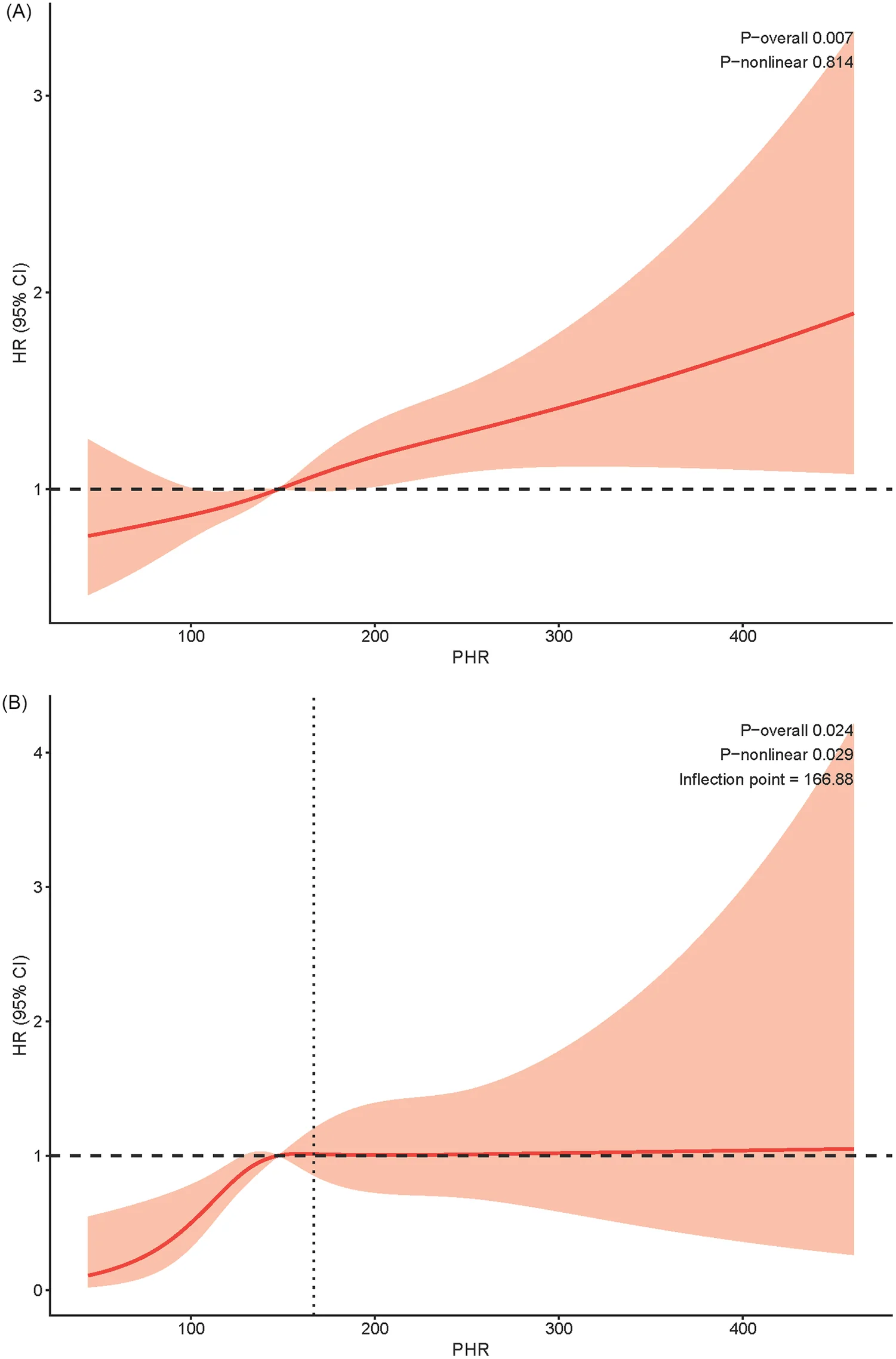
Dose-response relationships of PHR level with the probability of MACE and AMI. Restricted cubic spline analysis showed a significant overall association of PHR with MACE **(A)**, with no significant evidence of non-linearity (*P* for overall < 0.05; *P* for non-linearity > 0.05). For AMI **(B)**, both the overall association and the non-linear association were statistically significant (*P* for overall < 0.05; *P* for non-linearity < 0.05). The solid lines represent the estimated hazard ratios, and the shaded areas indicate the corresponding 95% confidence intervals. Models were adjusted for age, sex, current smoking, current drinking, hypertension, diabetes, antihypertensive medication use, hypoglycemic medication use, LDL-C, TG, BMI, and eGFR.

### Survival analysis

Kaplan–Meier curves for event-free survival are presented in Figure 3. According to the unadjusted log-rank test, participants in the third and fourth PHR quartiles had significantly higher cumulative incidences of MACE and AMI than those in the first and second quartiles (*P* < 0.05).

**Figure 3:**
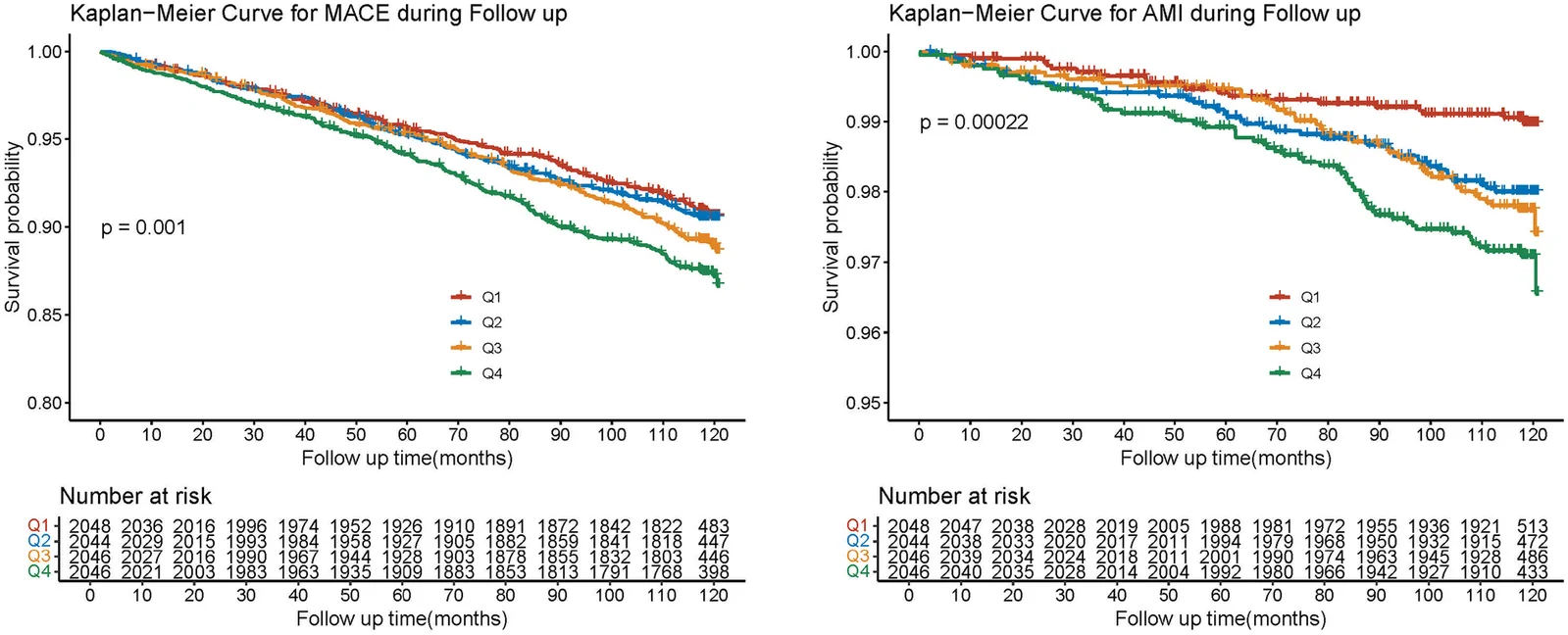
Kaplan–meier curves for survival probability, with follow-up in years.

### Subgroup analyses and interaction among variables

A statistically significant interaction was observed between PHR and drinking status in relation to MACE risk (*P* for interaction = 0.030). Among non-drinkers, participants in the highest PHR quartile had a higher risk of MACE than those in Q1 (HR: 1.70; 95% CI: 1.33–2.16). In contrast, this association was not evident among drinkers, for whom the HR for Q4 versus Q1 was 0.95 (95% CI: 0.66–1.37). After adjustment for multiple comparisons, no other stratification factors showed significant interaction with PHR in relation to MACE risk (Table 3). The subgroup-specific associations between PHR and MACE risk are further illustrated in the forest plot (Figure 4).

**Table 3:** Subgroup analysis of the association of PHR with MACE.

Subgroup	Hazard ratio (95% CIs)				*P* for interaction
	Q1	Q2	Q3	Q4	
Sex					0.272
Male	Reference	0.86 (0.64, 1.15)	0.96 (0.72, 1.28)	1.06 (0.80, 1.40)	
Female	Reference	1.12 (0.82, 1.53)	1.32 (0.97, 1.78)	1.57 (1.17, 2.12)	
Age, year					0.830
<65	Reference	1.15 (0.86, 1.54)	1.47 (1.11, 1.94)	1.75 (1.34, 2.29)	
≥65	Reference	1.03 (0.74, 1.43)	1.29 (0.92, 1.80)	1.83 (1.30, 2.57)	
Smoke					0.152
No	Reference	1.10 (0.86, 1.40)	1.31 (1.04, 1.67)	1.45 (1.15, 1.84)	
Yes	Reference	0.75 (0.48, 1.17)	0.75 (0.50, 1.15)	0.97 (0.66, 1.42)	
Drink					0.030
No	Reference	1.16 (0.90, 1.50)	1.44 (1.12, 1.84)	1.70 (1.33, 2.16)	
Yes	Reference	0.79 (0.54, 1.15)	0.78 (0.54, 1.14)	0.95 (0.66, 1.37)	
Hypertension					0.284
No	Reference	0.88 (0.60, 1.30)	1.03 (0.70, 1.51)	1.50 (1.05, 2.15)	
Yes	Reference	1.06 (0.82, 1.37)	1.13 (0.88, 1.46)	1.20 (0.94, 1.53)	
Diabetes					0.060
No	Reference	1.10 (0.84, 1.44)	1.25 (0.96, 1.63)	1.66 (1.29, 2.14)	
Yes	Reference	0.88 (0.62, 1.25)	0.99 (0.71, 1.39)	0.94 (0.67, 1.31)	
BMI, kg/m2					0.537
<28	Reference	1.00 (0.78, 1.26)	1.09 (0.86, 1.38)	1.41 (1.11, 1.78)	
≥28	Reference	1.08 (0.67, 1.76)	1.36 (0.86, 2.13)	1.31 (0.85, 2.03)	

Model adjusted for age, sex, smoke, drink, hypertension, diabetes, antihypertensive drug, hypoglycemic drug, LDL-C, TG, BMI, eGFR. The strata variable was not included when stratifying by itself.

BMI, body mass index; CI, confidence interval; HR, hazard ratio.

**Figure 4:**
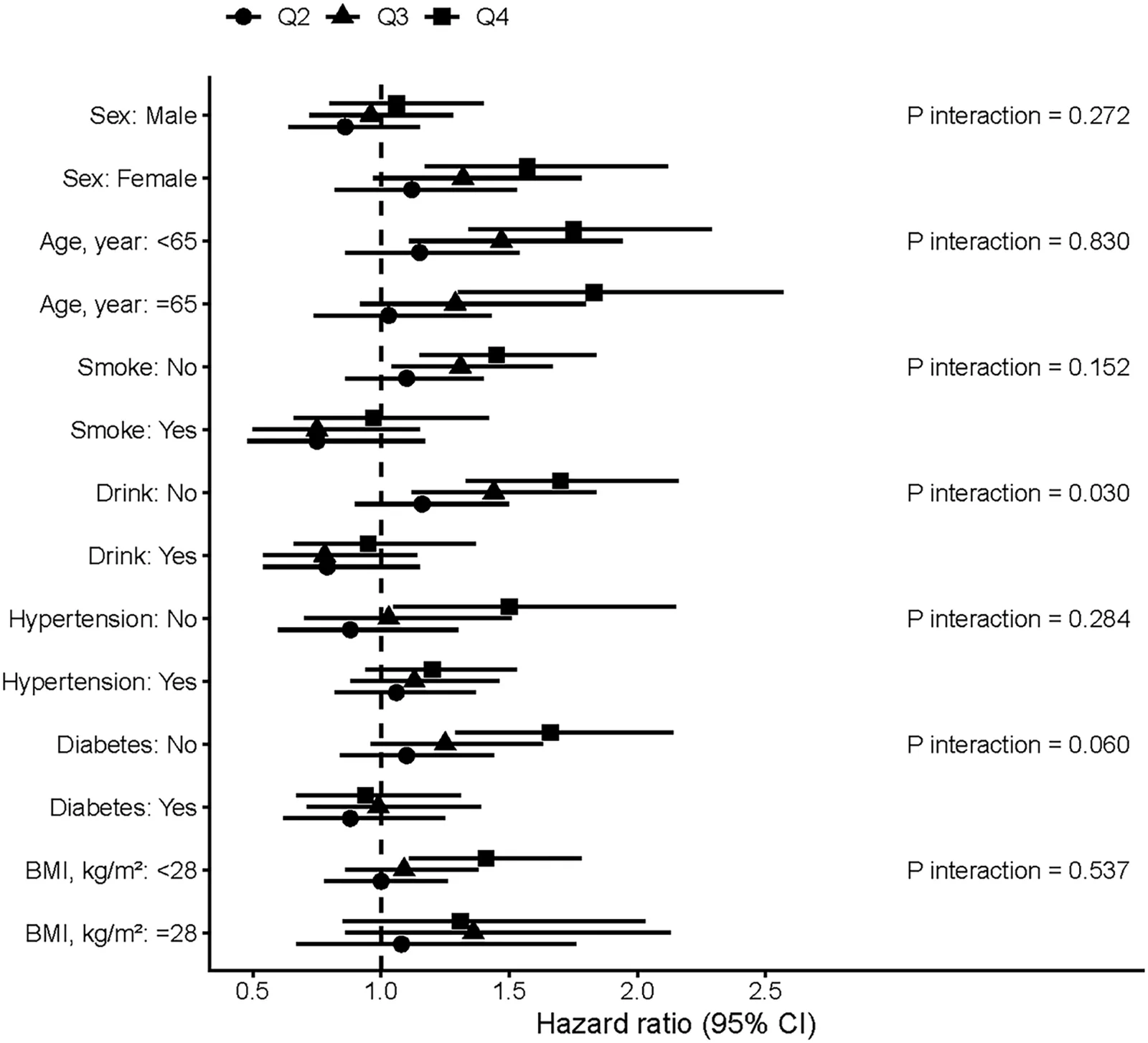
Forest plot of the association between PHR quartiles and MACE risk across predefined subgroups. Hazard ratios (HRs) and 95% confidence intervals (CIs) are shown, with Q1 as the reference group.

## Discussion

To our knowledge, this is among the first studies to examine the association between PHR and MACE in a community-dwelling population. In this large prospective cohort of Chinese adults, higher baseline PHR was associated with an increased incidence of MACE, with the association being particularly evident for AMI. By contrast, PHR was not significantly associated with the risks of stroke or cardiovascular mortality after multivariable adjustment. Of note, a modest increase in eGFR was observed across higher PHR quartiles at baseline. Because eGFR is an estimated measure derived from serum creatinine together with demographic factors such as age and sex, this difference may partly reflect differences in the demographic composition of the PHR groups rather than a direct association between PHR and kidney function. Therefore, this finding should not be interpreted as indicating better renal function associated with higher PHR.

HDL-C or platelet counts alone do not accurately reflect the body's metabolic disorders and inflammation. However, numerous studies in recent years have shown that PHR is an emerging biomarker for metabolism and inflammation (10, 11). Using NHANES data collected between 2005 and 2018, Zhang et al. (10) reported a significant association between higher PHR and stroke risk. In their analysis, the risk of stroke increased when PHR was above 223.684. However, this finding was not observed in our study, likely due to the fact that more than 80% of the participants in our cohort had a PHR value below 223.684. Furthermore, ethnic disparities between Chinese and American populations may also contribute to this difference. Ishwarlal Jialal et al. (11) conducted a retrospective analysis of 58 patients with nascent metabolic syndrome (MetS). On this basis, our study further demonstrated an association between higher PHR levels and an increased risk of MACE in a community-dwelling population, suggesting that PHR may serve as a potential biomarker associated with cardiovascular risk.

The biological mechanisms underlying the association between PHR and MACE remain to be fully clarified. We can explain the association in terms of atherosclerosis. Atherosclerosis is an important pathogenic mechanism for cardiovascular diseases, including MACE (12–15). HDL has been shown to demonstrate anti-atherosclerotic efficacy (16–18). Lower HDL-C concentrations may impair reverse cholesterol transport, leading to cholesterol accumulation in the arterial wall and favoring the development of atherosclerotic plaques (19, 20). Increased platelet counts may promote a prothrombotic state by enhancing coagulation activity and facilitating thrombus formation at injured vascular sites, which can further accelerate atherosclerotic progression. Elevated platelet counts also increase blood coagulability and contribute to thrombosis at sites of arterial injury (21, 22). Taken together, these mechanisms may partly explain the observed association between higher PHR and MACE.

### Strengths and limitations

This study has several notable strengths. To our knowledge, it is one of the largest prospective cohort studies to evaluate the association between PHR and subsequent MACE. Moreover, PHR can be readily obtained from routine blood test parameters, making it a simple, low-cost, and accessible potential marker, particularly in resource-limited healthcare settings.

Several limitations should also be acknowledged. First, because this was an observational study, causal inference cannot be established, and future multicenter prospective studies are warranted to validate our findings. Although sequential models with progressive adjustment for potential confounders were used and the associations remained generally consistent across models, potential overadjustment due to interrelated cardiometabolic and treatment-related covariates cannot be completely excluded. Residual confounding from unmeasured or incompletely measured factors may also remain. Furthermore, the proportional hazards assumption was not formally tested, and potential violations of this assumption cannot therefore be excluded. Second, the participants were recruited from a Chinese community-based population; therefore, whether the results can be generalized to other ethnic groups or populations requires further investigation. Third, PHR was assessed only once at baseline, which may not fully capture long-term changes in platelet count or HDL-C levels over time.

## Conclusion

In summary, findings from this large prospective cohort indicate that higher PHR levels were independently associated with increased risks of MACE and AMI among Chinese community-dwelling adults. These findings suggest that PHR may serve as a potential biomarker associated with elevated long-term cardiovascular risk.

## Data Availability

The data analyzed in this study is subject to the following licenses/restrictions: data will be made available upon reasonable request. Requests to access these datasets should be directed to lijianping03455@pkufh.com.
